# Naturalistic reinforcement learning

**DOI:** 10.1016/j.tics.2023.08.016

**Published:** 2024-02

**Authors:** Toby Wise, Kara Emery, Angela Radulescu

**Affiliations:** 1 Department of Neuroimaging, King’s College London, London, UK; 2 Center for Data Science, New York University, New York, NY, USA; 3 Center for Computational Psychiatry, Icahn School of Medicine at Mt. Sinai, New York, NY, USA

**Keywords:** reinforcement learning, decision-making, naturalistic, computational modeling

## Abstract

Humans possess a remarkable ability to make decisions within real-world environments that are expansive, complex, and multidimensional. Human cognitive computational neuroscience has sought to exploit reinforcement learning (RL) as a framework within which to explain human decision-making, often focusing on constrained, artificial experimental tasks. In this article, we review recent efforts that use naturalistic approaches to determine how humans make decisions in complex environments that better approximate the real world, providing a clearer picture of how humans navigate the challenges posed by real-world decisions. These studies purposely embed elements of naturalistic complexity within experimental paradigms, rather than focusing on simplification, generating insights into the processes that likely underpin humans’ ability to navigate complex, multidimensional real-world environments so successfully.

## Highlights

Real-world environments present a substantial challenge for human decision-makers due to their complexity, a characteristic that is often not replicated in reinforcement learning lab tasks.
Experiments that blend the control of lab tasks with the complexity of naturalistic environments have begun to identify processes that support naturalistic decision-making.
Humans use structure and regularity across naturalistic environments to enable effective and efficient decision-making in challenging and complex real-world situations.

## The need for naturalism

RL has emerged as a dominant framework for understanding the computational mechanisms supporting human learning and decision-making, allowing us to understand these processes in terms of algorithms for maximizing long-run reward. A wealth of research has leveraged this framework to understand varied facets of behavior, ranging from fundamental aspects of perception through to symptoms of mental health problems [1., 2., 3.]. The adoption of RL as a framework within computational cognitive science and computational psychiatry has arguably been central to many of the advances these fields have witnessed in recent years [4,5].

To date, RL researchers have typically sought to elicit specific human behaviors through carefully crafted tasks. These tasks distill the complexities of natural environments into simple paradigms, making complex decision-making processes amenable to lab-based characterization and explanation. This is a powerful approach that permits highly controlled examination of human learning and decision-making processes through a focus on their constituent parts. Tasks can further be designed to distinguish between competing hypotheses, formalized through computational modeling, providing tests of mathematically grounded hypotheses about learning and decision-making [6,7].

**Figure 1:**
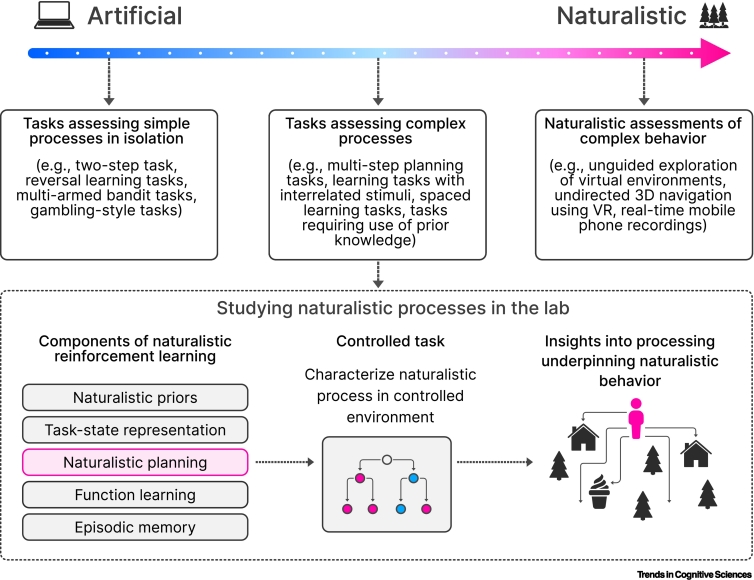
Research into naturalistic reinforcement learning can fall along a spectrum. We define naturalistic reinforcement learning as an approach that uses reinforcement learning as a paradigm to understand the complexity of naturalistic behavior, moving beyond simple tasks that eschew this kind of complexity. Research in this area can fall along a spectrum, ranging from traditional paradigms to fully naturalistic approaches that seek to replicate the complexity of the real world. In the middle lies the approach that most studies described in this article follow and which is described in the lower panel. A process that is relevant for naturalistic decision problems, and which is implicated in decision-making in the face of naturalistic complexity, is studied in the context of a more controlled laboratory task. Resulting insights can inform our understanding of naturalistic behavior. Abbreviation: VR, virtual reality.

**Figure 2:**
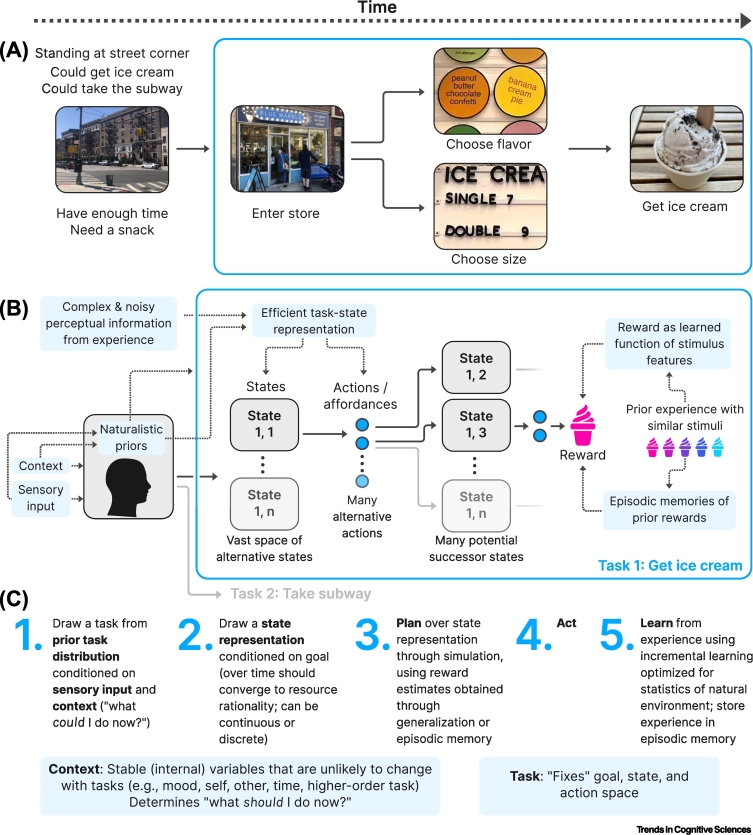
Illustrative example demonstrating the components of a real-world decision-making task. (A) The seemingly simple process of deciding to eat ice cream, where actions taken from a given starting state or context result in a desired outcome. (B) Schematic diagram demonstrating the complexity of this behavior and the ways in which naturalistic reinforcement learning processes can enable effective and efficient decision-making despite this complexity. The starting state depends on an interacting web of sensory input, broader context, and naturalistic priors. This leads to a ‘task’ that comprises myriad states and potential actions to be evaluated, but which can be represented efficiently to facilitate effective decision-making despite the true complexity of the problem. Actions must be evaluated based on long-run rewards that are estimated based on knowledge gained from previous experience. (C) The step-by-step process of deciding in a naturalistic environment. First, a task must be chosen, followed by an efficient representation of the task’s component states, which depends upon context and goals. Planning proceeds based on this task state representation, using reward estimates derived from sparse prior experiences. Finally, upon acting and receiving a reward, learning occurs in a way that is optimized for the statistics of the natural environment.

This approach does not capture the complexity of the real world; rather, simplification is the goal. It can be argued convincingly that the reductionism implicit in this traditional approach, both in the design of task environments and computational models, is necessary to understand such complex processes; by understanding the constituent parts in isolation, we can gradually build towards a complete understanding of the whole system. Here, we survey research that suggests that we have much to gain from taking a broader view of human behavior that moves beyond a focus on isolated, unnaturalistic elements of learning and decision-making. If our goal is to understand real-world human behavior, this work suggests that we should begin to embrace its complexity, adopting naturalism alongside reductionism. We refer to this as ‘naturalistic RL’. We note that ‘naturalistic’ can have different connotations; here we use the term to refer to RL approaches that purposely integrate the complexity inherent in naturalistic environments into both theory and experimental design (Figure 1, Figure 2).

Naturalistic RL encompasses a broad range of approaches (Figure 1). Fully naturalistic methods, using entirely unconstrained ‘tasks’, represent the extreme end of this approach [8,9]. However, a great deal can be learned from tasks and models that purposefully integrate elements of natural complexity into their design, blending a naturalistic view of human behavior with the reductionism of traditional experimental paradigms. In this article, we summarize recent research across this spectrum, outlining how a more naturalistic approach has begun to shine a new light on key aspects of human learning and decision-making. This area of research aligns with other efforts to characterize naturalistic behavioral patterns, such as computational ethology [8., 9., 10.], but focuses specifically on applying RL as a framework for understanding the mechanisms underpinning such behavior.

A common theme that emerges from this body of work is the notion of structure: representing and using patterns in naturalistic environments that can make decision-making practicable in the face of complexity. We demonstrate this by working through different elements of realistic decision problems, progressing from high-level expectations about the environment through to lower-level components such as reward. We illustrate how each element can become complex when considered within naturalistic environments and highlight how recent research has demonstrated how humans exploit structure and regularity to solve naturalistic decision problems.

## Naturalistic priors and context

Decisions in the real world typically depend on information we observe or learn before we make a choice. Contexts and information sources are often shared across decisions. While these commonalities may be obscured by the noise inherent in complex natural environments, inferring, representing, and leveraging the statistical structure of our experiences can enable more efficient and accurate decision-making. A wealth of work points to the ways in which priors influence and aid human perception and decision-making (e.g., [11., 12., 13.]).

However, tasks typically used to study human RL often intentionally prevent prior knowledge from being useful by designing stimuli and tasks that are both novel and simple (e.g., fractal stimuli) and by eliciting isolated, repeated, and independent decisions removed from any shared context. Although these tasks provide valuable insights into human decision-making, they often do not tell us how priors are represented and used, especially in conditions that better approximate the real world.

In natural environments, humans bring a rich space of candidate object features and interactions to each task. For example, when I see a red, shiny, round, and small item from a distance, I might expect it to be an apple. If I am hungry, I might also decide to retrieve it. I already have the labels for these features (e.g., red) and potential actions (e.g., retrieve) before this encounter and, at least in part, from prior experience. Compared with the simple objects often used in reduced laboratory settings (e.g., the green stimulus), the features humans use to represent naturalistic objects are often highly multidimensional and range from concrete (e.g., round) to abstract (e.g., royalty) [14,15]. Furthermore, naturalistic objects have characteristic affordances that influence the link between specific environmental states and their potential actions. Thus, prior interactions with objects might constrain the set of potential actions brought to a task in the context of RL, as demonstrated by recent work across both artificial intelligence (AI) and cognitive science [16., 17., 18.].

Real-world scenes also have typical object arrangements, which can inform decision-making. When I walk into a living room and see a sofa, I may expect to see a television in front of it and a coffee table nearby. These scene semantics have been shown to shape the way we interact with, attend to, and remember objects and scenes [19]. Spatial priors based on these naturalistic scene semantics guide search to scene regions meaningful for the agent’s task [20] and make search more efficient generally [21,22]. Studies show that people construct accurate and detailed memories of real-world scenes, with heightened recall for objects in visually salient and meaningful regions [23], especially when they are relevant to achieving a goal [22,24].

Naturalistic priors may also act at a more algorithmic level, guiding the decision-making process itself based on expectations about the structure of the environment. For example, a recent study demonstrated how providing story-like versus abstract instructions for a traditional two-step task led to more model-based decision strategies. This suggests that providing prior knowledge about environment structure, specifically using familiar objects and actions, makes participants more likely to leverage this information in their decision-making [25]. Recent work also demonstrates how contextual factors, which may include those derived from prior experience, can induce biases in decision-making [26,27] and learning [28]. Related work also demonstrates how learned strategies can generalize across related tasks [29], providing a mechanism that could underpin efficient learning in naturalistic environments where behavior must be optimized across similar scenarios. By leveraging prior knowledge and experience, learning and decision-making can be optimized for the specific task at hand.

Together, this growing literature emphasizes the centrality of multidimensional, abstract, and naturalistic priors as a form of structure that enables effective and efficient decision-making in natural environments. Approaches that account for and leverage these priors within RL models could generate intriguing insights into how humans approach real-world decision problems that could not be gleaned from more constrained approaches.

## Task-state representation

**Figure 3:**
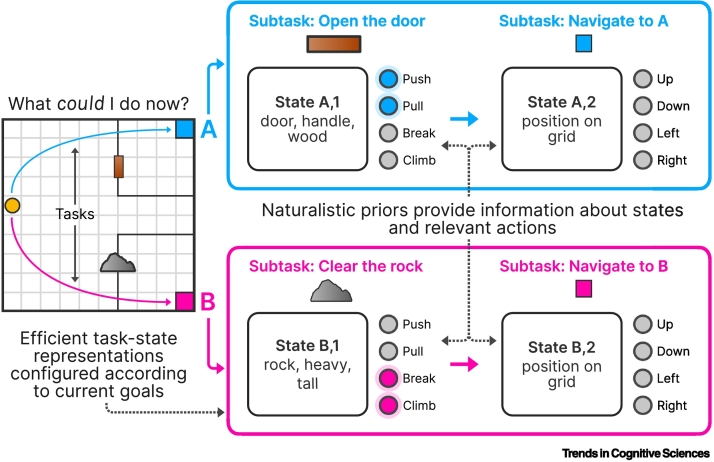
An illustrative example of task-state representation with the influence of naturalistic priors. In this example, the agent (yellow dot) is in an environment with two potential tasks, ‘get to A’ or ‘get to B’, each with potential subtasks depicted in the blue and pink outlined boxes. For example, to get to A, the agent must enter a room, which requires opening a door. Tasks can be represented in an efficient manner that depends upon the agent’s goals [37], informed by naturalistic priors. The agent might be able to leverage naturalistic priors [16,21], which include labels for actions (push, pull), objects (door), and contexts (wall) from previous experience, to help create a useful state representation and constrain relevant actions for the given task or subtask. As a result, the agent will be able to limit the action space to only the most relevant actions for a given subtask, such as pushing and pulling for the first blue subtask (‘open the door’) or to breaking and climbing for the first pink subtask (‘clear the rock’), while representing a broader set of actions as suggested by relevant naturalistic priors (e.g., in the case of the final subtasks, ‘navigate to A’ and ‘navigate to B’).

Naturalistic priors provide structure that enables us to interpret and understand our environment. However, effective decision-making also necessitates a representation of the decision problem at hand (Figure 3). Task-state representation is often studied through the lens of cognitive maps [30]. In this view, a representation of a task is defined as a map of discrete interconnected states, which an agent traverses as it learns to maximize reward. The exact structure of this map depends on the features being considered for representation in the first place [31]. For instance, thinking about a museum in terms of spatial features might elicit a map of rooms, while considering artistic styles would elicit a map of individual artists. An optimal task-state representation may be a conjunction of multiple features [32]. Learning and decision-making in the real world necessitate the additional step of representation learning, whereby features of the environment are represented, integrated into a cognitive map, and modified with experience [33,34]. For example, acting in a social situation may benefit from representing the trustworthiness, power, or generosity of others [35,36].

Recent resource-rational accounts propose that representations are selected to be simple and useful, in the sense that they significantly change the best course of action when planning [37]. This resource-rational view of representation provides a principled reason for why people learn to balance abstraction and detail as they gain experience with new tasks [38]; through focusing on underlying abstract structure and ignoring details that are irrelevant for determining the best policy, simple and useful task representations can be constructed. Such abstractions are thought to be supported by the hippocampus [39,40], being maintained through hippocampal replay [41,42], and likely play a crucial role in providing simple and useful structure across complex naturalistic tasks. Abstraction can also occur temporally, for example, through compressing multistep action sequences into a single ‘option’ [43].

But how might such an abstraction process be realized in naturalistic contexts? One possibility is that goals themselves impose constraints on the representations to be considered. For instance, an aesthetic judgment of a visual stimulus may spontaneously elicit a different set of relevant features than those needed to navigate social situations [44]. Such goal dependence is obscured in classic reward-maximization paradigms, which do not explicitly alter the meaning of reward and value as a function of arbitrary goals [45., 46., 47.].

How can we better understand the process by which goals themselves are specified? Work in the AI literature has suggested that goal specification in terms of language is an especially effective way to train RL agents to solve complex tasks [48]. Language makes it possible to express high-level goals compactly (‘make dinner’); encode candidate policies as multistep sequences of states and actions (‘at 6 pm, defrost chicken, at 6:30 pm, cut veggies’); compute predictive summary statistics of such policies on the fly (‘defrosting chicken often means tacos for dinner’); and represent policies hierarchically, at a level abstraction that optimizes transfer and reuse (‘cook tacos’). In other words, language has many of the properties that are critical for efficient task representation [31,37,43,49., 50., 51.]. In line with this proposal, recent work in human RL has begun to reveal that linguistic information can improve learning performance, especially as the complexity of the task increases [52., 53., 54., 55.].

Taken together, these results suggest a role for language in dynamically specifying goals and representing the environment to efficiently accomplish them. Future study of how language interacts with learning and decision-making will likely benefit from behavioral assays in which the structure of naturalistic environments can be more readily quantified [56].

## Planning in large and complex state spaces

Effective task-state representations provide a basis for planning. However, task-state representations can easily become large and complex in naturalistic environments, even when naturalistic priors are used to simplify representations, making planning itself a challenge.

**Figure 4:**
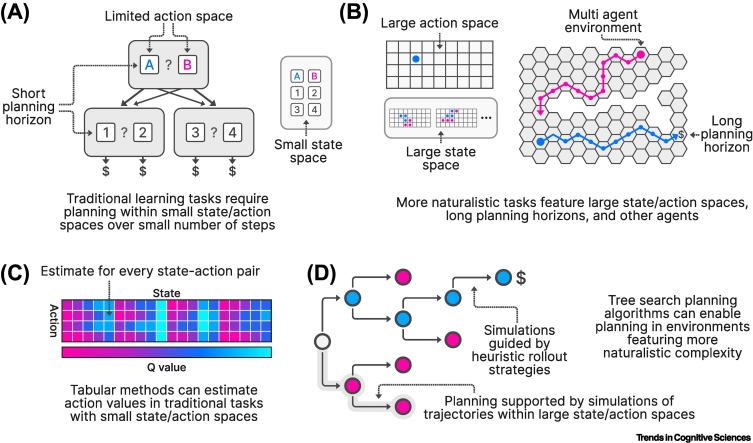
Naturalistic planning. (A) Traditional planning tasks require participants to plan a small number of steps into the future within a small state space, using a limited range of possible actions (e.g., choosing left or right). (B) More naturalistic tasks incorporate large action and state spaces, better approximating the complexity of real-world environments. They also require participants to plan further into the future and can incorporate other agents to better reflect the need for long-term (and potentially social) planning in real-world environments [72,73,75]. (C) Tabular planning methods determine the optimal policy for a given decision problem by estimating the value of every action in every state, making them highly effective for problems with a small set of states and actions. (D) Real-world decision-problems often feature far larger state and action spaces, rendering tabular methods impractical. Planning algorithms that rely on simulations of state-action trajectories (such as tree search planning algorithms) fare better in these circumstances, permitting effective action selection where tabular methods fail [72,73,75].

Traditional dual systems accounts posit that humans possess two decision-making systems: a quick, computationally simple system that learns action values through direct trial-and-error experience, and a slow, computationally demanding system that estimates action values using an internal model of the world [57]. These are typically referred to as the ‘model-free’ and ‘model-based’ systems, respectively, and humans have been shown to flexibly deploy each system depending on the expected success of each strategy [58., 59., 60.]. The majority of the literature in this area has used elegant, but simple, task designs to disentangle the contributions of these systems, the most commonly used being the ‘two-step’ task [57,58] (Figure 4A). However, while these tasks are powerful in their ability to tease apart the contributions of distinct systems, we might question how well they approximate real-world decision-making.

Real-world state and action spaces are typically large, even when reduced through efficient task-state representation (Figure 4B). Transition structures are often nondeterministic, or even unknown entirely. Finally, real-world decision problems often involve a social element; many of our day-to-day decisions will involve other people [61,62,63]. Recent studies have begun to directly address these elements of real-world decision problems. Efforts to understand how model-based and model-free strategies are engaged in social situations have revealed that the model-free system is differentially engaged when learning to avoid pain for oneself or another [61], demonstrating that these two systems are deployed differently depending on social context. A similar approach has been used to understand how human observers arbitrate between model-free and model-based strategies when attempting to predict others’ actions [64], further emphasizing how this basic principle can be applied to decision problems that account for complexities of the real world.

The question of how human decision-makers determine the best course of action in expansive state spaces with complex transition structures remains a largely open one, but recent studies have begun to make some inroads. A prominent theme within this work is a move from straightforwardly optimal strategies to simulation-based strategies that rely on heuristics to reduce computational cost [49] (Figure 4C). Tree search planning algorithms represent an approach to planning that scales to vast state spaces [65] and may better describe human planning in the real world (Figure 4D). By simulating trajectories of potential actions and outcomes in a principled manner, these algorithms can approximate the optimal policy in situations where traditional model-based algorithms are computationally infeasible. While the use of tree search models in research into human decision-making is not especially new [66., 67., 68., 69.], recent advances in model-fitting [70,71] have permitted their use in explaining human behavior in increasingly large state spaces that better approximate real-world decisions.

One recent study [72] demonstrated that human decision-making in a chess-like game, with a large state space and high branching factor, is well explained by a tree search planning algorithm that simulates trajectories of potential actions and outcomes. A similar modeling approach was used in another study to model avoidance behavior in a complex virtual environment [73], further showing that tree search models can explain human behavior in tasks that better approximate real-world decision problems. Notably, this task also incorporated a predator agent whose actions were incorporated into the tree search simulations, highlighting how this approach can incorporate social information into the decision-making process. Another recent study further exploited this approach to demonstrate that individuals differ in the subjective cost of planning, explaining variability across individuals in planning depth [74]. Providing further support for the utility of this type of planning strategy in naturalistic environments, one study showed through simulation that tree search planning algorithms are especially advantageous in large, open environments where the agent has an extended visual range, characteristics of many of the terrestrial environments that humans inhabit [75].

Tree search algorithms are powerful because they allow for efficient searching of the state space. They often rely on some form of heuristic to guide this search and it is possible that such heuristics are key to the success, flexibility, and efficiency of human planning in naturalistic settings. Heuristics can be used to approximate the value of particular states, for example, board states in a board game [72,76], providing a quick and reasonably accurate estimate of state value that can be used to guide planning. There is also a rich literature on the role of heuristics in guiding the planning process itself, for example, by determining when branches in a decision tree should be ‘pruned’ [67,68,76].

Recent work has taken a principled approach to the discovery of new heuristics, for example, seeking to elucidate the ways in which humans jointly maximize both reward and computational efficiency with the framework of resource rationality. One such study showed that human planning is broadly similar to that of an optimal model designed to balance reward against computational cost [77], suggesting that human planning has developed to maximize efficiency in the face of limited computational resources. In contrast, combinations of existing known heuristics mimicked human planning less accurately, suggesting that there are subtle aspects of human planning that are driven by a need to reduce cost.

The nature of human planning in more real-world environments is only beginning to be characterized, but future work using naturalistic approaches will likely uncover further simplifying heuristics that provide for efficient planning by exploiting regularities across complex environments.

## Sparse and sequential rewards

Ultimately, the goal of any RL agent is to maximize reward. However, even something as seemingly simple as reward can become challenging to estimate accurately in naturalistic environments.

**Figure 5:**
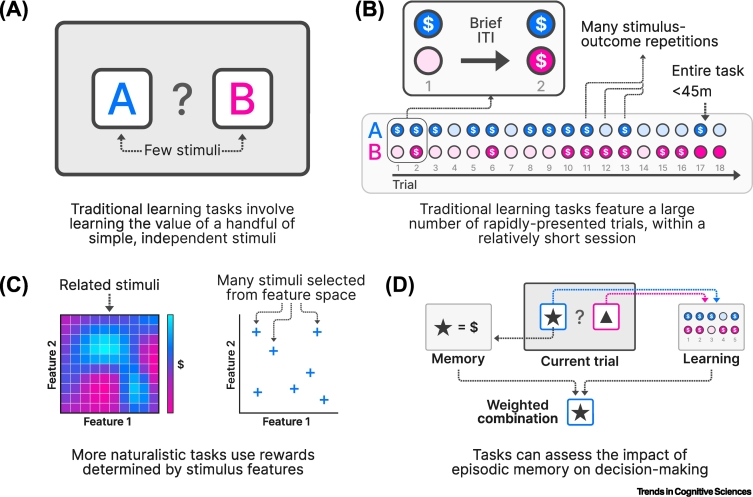
Sparse and sequential rewards. (A) Traditional learning tasks require participants to learn the value associated with a small number of stimuli or actions. These stimuli are typically unrelated (i.e., it is not possible to generalize value from one to the other based on shared properties). (B) In traditional tasks, stimulus–outcome pairings are shown repeatedly, typically in quick succession, over a single relatively brief session. (C) In more naturalistic tasks, stimuli may be interrelated based on shared features, with their value depending on these features. This better approximates the complexity of real-world learning, including the need for generalization across similar stimuli [92,97,99]. (D) Tasks can also be designed to test the influence of episodic memory, a system that is likely to be important in real-world learning [86]. Abbreviation: ITI, inter-trial interval.

Research into human reward estimation has learned much from traditional learning paradigms. These follow a common theme: participants learn the value of a small set of independent stimuli based on repeated presentations (e.g., [57,78], Figure 5A). Such tasks have advantages: they are simple, straightforward to administer and analyze, and produce behavior that is well described by simple models [7]. However, in the real world, learning opportunities are typically sparse; to learn whether I like a restaurant, I am unlikely to visit hundreds of times in quick succession. In contrast, an assumption inherent in many traditional tasks is that value is determined according to a running average of its associated reward, estimated through incremental trial-by-trial learning (Figure 5B). The sparse nature of real-world experience may necessitate alternative strategies.

One strategy that has been shown to support real-world learning is the use of episodic memory [79., 80., 81.]; drawing on specific prior experiences can be effective in the absence of incrementally learned value. Humans are thought to retrieve episodic memories to guide value-based decision-making, as evidenced by hippocampal involvement in even simple decision problems [82,83]. Further evidence comes from the finding that reminders of specific prior experiences can influence value-based decisions [84,85], suggesting that reactivating episodic memories influences decision-making. The contributions of incremental learning and episodic memory retrieval also appear to be weighted adaptively according to uncertainty in the predictions of each system [86].

Even when incremental learning is possible in the real world, however, it will often occur over the course of days or weeks rather than seconds or minutes. This is not a trivial distinction; distinct neural and computational systems are involved depending on the timescale, with hippocampal involvement being prominent when learning experiences are spaced apart [87,88]. In contrast, traditional learning tasks are known to be highly working-memory dependent [89., 90., 91.], suggesting that these tasks may be tapping into underlying systems that are distinct from those used to guide real-world decisions. Of note, as well as being more naturalistic, spaced learning is more similar to paradigms typically used in animal research and such paradigms in humans provide for more straightforward comparisons across species.

Incremental learning is also typically quantified using models that overweight recent experience, where real-world learning may benefit from alternative strategies. One study showed that subjective mood during a gambling task is better described by a model that over-weights early experiences [92]. Notably, this effect was especially pronounced when the task structure is designed to better reflect the statistics of real-world experiences (i.e., when there is structure in the outcomes received, as opposed to being entirely randomly selected), suggesting that more naturalistic environments may prompt reliance on alternate learning strategies.

A further aspect of real-world learning often absent from traditional paradigms is the interrelated nature of learning experiences. If I enjoy the food of a particular cuisine at one restaurant, I can infer that I may enjoy other restaurants serving the same cuisine. One approach to studying the role of generalization in learning is to model human behavior in ‘correlated bandit’ tasks, a variant of a multi-armed bandit where arms are correlated according to a predefined covariance function. Humans use this covariance structure to infer the values of previously unchosen bandits, as revealed by computational modeling demonstrating substantially better fit of models incorporating generalization relative to independent learning models across a number of studies [93., 94., 95., 96., 97., 98.]. Further work has extended this to stimuli whose value is computed as a function of multiple perceptual features, demonstrating that humans can infer value by combining limited direct value learning with sophisticated function learning [99], as opposed to using stimulus-specific incremental learning.

This growing literature emphasizes the importance of understanding how humans use structure and regularity to determine expectations of reward in complex environments. Future efforts exploiting less constrained paradigms may provide further insights into how humans learn about and infer rewards in real-world environments.

## Naturalistic computational psychiatry

Real-world relevance becomes especially crucial when drawing inferences regarding how learning and decision-making processes can go awry, as is the case across many mental health conditions [2,5,100]. If we wish to make a difference to the lives of people experiencing these conditions, it is imperative that our findings extend beyond the lab. This issue is one that has already received attention (see [101] for a thorough discussion), but the emerging research discussed here highlights further avenues for more naturalistic computational psychiatry research.

To date, most studies in the field have used traditional paradigms [5], although efforts have been made to evoke more naturalistic behavior by using gamified variants of these tasks that move beyond simple choices between unnaturalistic stimuli (e.g., [102]). Fewer efforts have sought to investigate the involvement of the more complex, naturalistic learning and decision-making processes outlined in this article, but we believe such research could be highly valuable for the field. Given the common thread throughout naturalistic RL of inferring, representing, and using structure in a complex and noisy world, this approach could reveal how and why many mental health problems are associated with altered representations of the external world.

Planning serves as a useful example of how a naturalistic approach may prove fruitful. A large body of research has highlighted that model-based planning is impaired across diagnoses, with more recent research using a process termed ‘computational factor modeling’ revealing that this impairment is specifically linked to transdiagnostic symptoms of compulsivity and intrusive thought [5,103]. While robust, this finding has emerged from traditional tasks that focus on two-step planning problems, using simple models of model-based planning. As described previously, more recent efforts have sought to understand the nature of model-based planning, moving beyond a focus on the extent to which it is used. While experimental work in this area is yet to emerge, intriguing theoretical and modeling work suggests that alterations in these more complex planning processes may be linked to symptoms such as worry [104., 105., 106.], which can be viewed as negatively-focused planning [104].

## Concluding remarks

Naturalistic RL has the potential to change how we think about human learning and decision-making by focusing on understanding how we make effective decisions in situations that more closely resemble the real world. The research surveyed here demonstrates how studies have begun to systematically explore how humans make effective decisions in environments that incorporate elements of naturalistic complexity.

A common theme throughout this work is the notion of structure. The natural world is large, complex, and noisy and successful decision-making relies on building representations of its latent structure and using them in an efficient manner. This fundamental principle plays a central role across diverse aspects of naturalistic decision-making, ranging from the use of features to guide generalization when learning in a multidimensional world, through to the exploitation of language as an efficient way to represent complex decision problems. Although beyond the scope of this review, naturalistic structure is also likely to vary across individuals and culture, a challenge RL researchers will increasingly need to confront as their models attempt to explain more naturalistic behavior. Overall, by focusing on the complexity of naturalistic environments, the research reviewed here forces us to think more deeply about the foundations of the traditional RL paradigm; even something as seemingly simple as a ‘task’ can be difficult to define in the context of naturalistic complexity.

Another caveat that applies to much of the work discussed here is that complexities inherent in defining a ‘reward’ often appear underappreciated and underexplored. Many approaches assume that participants’ subjective reward functions align perfectly with those implied by the task, with reward functions typically being treated as fixed while other aspects of the task-state representation are reconfigured to optimize behavior in the face of real-world complexity. However, it is possible that flexible and efficient responses to naturalistic complexity may also emerge through the development of reward functions in which relevant knowledge about the task is embedded. This approach has been shown in AI research to produce general behavioral patterns, such as exploration, that are useful across a diverse range of tasks [107].

While even in ‘non-naturalistic’ tasks, there are elements of naturalism which may not have been previously considered (Figure 1 [25]), the majority of the work we have discussed sits at a crossroads between traditional paradigms and fully naturalistic approaches, using experimental designs that follow a largely reductionist approach to address elements of naturalistic complexity. This has resulted in a significant shift in our understanding of how humans solve naturalistic decision problems and many interesting unanswered questions (see Outstanding questions), highlighting the versatility of RL as a framework that can guide inquiry across environments of varying complexity.

Nevertheless, a fully naturalistic RL approach will be challenging to implement, and may depend on further theoretical and methodological advances (Box 1). This raises an important practical question: when are more naturalistic approaches preferable?

Box 1 **Methods for assessing naturalistic RL**

The study of naturalistic RL has capitalized on advances in a number of methods.

Virtual environments: More naturalistic scenarios can be created by using relatively open 2D or 3D environments that better reflect the qualities of real-world environments (e.g., having large state spaces and other agents that behave in complex ways). Modern software (e.g., Unity) and programming languages (e.g., JavaScript) allow for 2D or 3D environments to be developed and even deployed over the web with relative ease.

Virtual reality: Many aspects of naturalistic RL could be best assessed by immersing the participant within a virtual environment. Virtual reality provides a way to do this in a highly effective manner with highly customizable and immersive environments, while also providing for the measurement of complex actions (such as reaching) [108,109].

Portable recordings: New developments in mobile phone tracking and measurement capabilities as well as other portable recording devices offer the opportunity to collect a wide variety of real-time, ecological, and momentary assessments. These include measurements such as heart rate, GPS, electroencephalography, learning tasks, and mood assessments, all of which could be captured as participants navigate the natural world [110].

Neural networks: Advances in deep learning in recent years have enabled the development of tools that can automatically extract features of behavior in a purely data-driven way [111]. Some work has already begun to apply these tools to human learning and decision-making, whether in standard experimental tasks [112] or less constrained video games [114], but their combination with truly naturalistic paradigms could lead to even greater insights into these processes.

Flexible model-fitting approaches: Complex behavior necessitates complex modeling approaches, but this has historically been challenging to the limitations inherent in traditional model-fitting methods. Modern model-fitting methods such as simulation-based inference [70,114] allow for computationally demanding and stochastic models, which can better describe processes like planning, to be fit with minimal computational burden.

Alt-text: Box 1

While clearer guidelines will emerge with more research, we suggest the following criteria for when to consider methods that afford a more naturalistic approach:

1. When the researcher is interested in studying how processes relevant for decision-making interact and has *a priori* reason to believe the interaction is meaningful for behavior. For example, it would not have been possible to reveal that efficient representations of the environment are key to successful planning without studying planning in tasks incorporating naturalistic complexity [37,72].
2. When measuring latent representations of the stimulus is key to answering the question at hand. For example, understanding which features are important in driving decisions about where to look requires understanding the structure of naturalistic environments [108].
3. When interested in individual differences, particularly in the context of special populations. For instance, recent work has shown that, in adolescents, the degree to which model-based parameters generalize across tasks can vary [28]. To the extent that such variation arises from variability in stimulus interpretation, naturalistic approaches may offer an experimental tool for studying that process and integrating it with the study of learning and decision-making.

We are optimistic that the work outlined in this article represents only the beginning of a significant shift within human RL towards an approach that can more fully characterize decision-making in the real world.

Outstanding questions What are the naturalistic priors that people consider when selecting tasks in the real world? We have a multitude of prior experience to draw on when selecting tasks; how do we decide which priors should be applied in the current situation?

How do people segment tasks in continuous environments? Tasks are not always discrete entities and can instead be defined according to continuous characteristics; how can we segment these?

What are the intrinsic reward signals that enable task segmentation and spontaneous behavior? Some aspects of behavior are likely dependent upon intrinsic reward signals, but these are yet to be fully understood.

How do people specify tasks with arbitrary goals? Humans are able to flexibly determine the appropriate current task depending on various types of goal; how is this achieved?

How does internal context (e.g., affect, interoception) influence decision-making? There are numerous studies investigating contextual influences on decision-making, but this is likely also dependent on internal states.

Which heuristics guide planning and how are they selected? Humans likely exploit numerous heuristics to guide planning in naturalistic environments, but these may be selected according to the structure of the decision problem at hand.

How can we adapt existing RL models to fully naturalistic environments that do not include trials and decision points in the typical sense? Many of the models typically used in RL research depend on simplified task structures and may need to be modified substantially to be used in unconstrained environments.

How do reward functions adapt to incorporate knowledge about naturalistic environments? Many approaches described here treat reward functions as fixed, when they may instead adapt to deal with naturalistic complexity. More naturalistic approaches may be useful in characterizing the reward functions used by humans in real-world environments.

How can naturalistic RL build links between human and animal research? In many ways, animal research has made greater progress in developing naturalistic assessments, for example, through the use of virtual reality [115] and automated behavioral labeling [111]. Conversely, human naturalistic RL may lead to the development of models that can better explain animal behavior.

Alt-text: Outstanding questions
